# Association of UHR and ECG parameters with type 2 diabetes mellitus in non-alcoholic fatty liver disease

**DOI:** 10.3389/fendo.2025.1643842

**Published:** 2025-08-28

**Authors:** Yaping Wang, Mingyan Liu, Wei Li, Yaping Liu, Xiaowei Tang, Fei Sun, He Zhu, Xiangming Tang

**Affiliations:** ^1^ Department of Electrocardiogram, Yangzhou Wutaishan Hospital of Jiangsu Province, Teaching Hospital of Yangzhou University, Yangzhou, China; ^2^ Department of Ultrasound, Yangzhou Wutaishan Hospital of Jiangsu Province, Teaching Hospital of Yangzhou University, Yangzhou, China; ^3^ Department of Clinical Laboratory, Yangzhou Wutaishan Hospital of Jiangsu Province, Teaching Hospital of Yangzhou University, Yangzhou, China; ^4^ Department of Psychiatry, Yangzhou Wutaishan Hospital of Jiangsu Province, Teaching Hospital of Yangzhou University, Yangzhou, China; ^5^ Department of Thoracic Surgery, The Affiliated Taizhou People’s Hospital of Nanjing Medical University, Taizhou School of Clinical Medicine, Nanjing Medical University, Taizhou, China; ^6^ Phase I Clinical Research Center, Taizhou School of Clinical Medicine, Nanjing Medical University, Taizhou, China; ^7^ Department of Neurology, The Affiliated Taizhou People’s Hospital of Nanjing Medical University, Taizhou School of Clinical Medicine, Nanjing Medical University, Taizhou, China

**Keywords:** non-alcoholic fatty liver disease, diabetes mellitus, uric acid to high-density lipoprotein cholesterol ratio, electrocardiogram parameter, biomarker

## Abstract

**Background:**

Non-alcoholic fatty liver disease (NAFLD) is the most prevalent liver disease globally. NAFLD increases the risk of type 2 diabetes mellitus (T2DM) while lacking clinical predictors. This study aims to investigate the characteristics and clinical significance of the uric acid (UA) to high-density lipoprotein cholesterol ratio (UHR) and electrocardiogram (ECG) parameters in NAFLD patients, both with and without T2DM.

**Methods:**

We compared 102 NAFLD with T2DM (NAFLD-T2DM) cases to 113 NAFLD without T2DM (NAFLD-nT2DM) cases. Baseline data and biochemical indicators, including UHR, were collected and analyzed in each group. A 12-lead ECG was used to collect parameters that were compared between the two groups. Multivariate logistic regression analysis was employed to identify factors influencing NAFLD with T2DM. Receiver operating characteristic (ROC) curves were utilized to assess the clinical value of UHR combined with ECG parameters in identifying T2DM risk among NAFLD patients.

**Results:**

Compared to the NAFLD-nT2DM group, the NAFLD-T2DM group exhibited significantly higher levels of triglycerides (TG), fasting plasma glucose (FPG), UA, and UHR, while aspartate aminotransferase (AST) levels were lower (P < 0.05). The incidence of ST-T changes, heart rate, and P wave duration was also higher in the NAFLD-T2DM group, whereas the QT interval was shorter (P < 0.05). Multivariate logistic regression analysis revealed that UHR, ST-T changes, heart rate, QT interval, and P wave duration are independent factors associated with the incidence of T2DM in NAFLD. ROC curve analysis indicated that the area under the curve (AUC) for the combination of five variables in predicting T2DM in NAFLD was 0.949 (95% CI: 0.905-0.977, P < 0.05), with a sensitivity of 91.96% and a specificity of 93.55%, significantly superior to those of individual indicators.

**Conclusion:**

UHR and ECG parameters are associated with T2DM in NAFLD patients. The combination of UHR and ECG parameters demonstrates predictive value for the incidence of T2DM in NAFLD patients. Clinical attention should be directed toward the levels of UHR and ECG parameters in NAFLD with T2DM.

## 1 Introduction

The advancement of the social economy, improvements in living conditions, and lifestyle changes increase the incidence of multiple chronic diseases, including hypertension, diabetes, and non-alcoholic fatty liver disease (NAFLD). NAFLD is defined as a clinical syndrome primarily characterized by the accumulation of fat in hepatocytes when excluding alcohol consumption and other contributing factors (1). Approximately 25% of adults worldwide are affected by NAFLD (2), with prevalence in Asia ranging from 15% to 40% (3). Studies suggest that by 2030, more than 300 million individuals in China, over 100 million in the United States, and 15 to 20 million in major European countries will be affected by NAFLD (4). As the most common chronic liver disease, NAFLD may also play a significant role in the progression of chronic liver disease to cirrhosis and liver cancer (5). The primary pathological features of NAFLD include hepatocellular edema, steatosis, inflammation, and varying degrees of hepatic fibrosis (6). The pathological change of NAFLD is partially reversible, therefore, early disease management is crucial for preventing and halting the progression of chronic liver disease to more severe stages, such as cirrhosis and liver cancer. Despite significant progress in understanding the pathogenesis of NAFLD, effective therapies remain limited in clinical practice (7). Consequently, enhancing the prevention and managing comorbidities are particularly important in the clinical approach to preventing and treating NAFLD.

NAFLD frequently coexists with various comorbidities, particularly type 2 diabetes mellitus (T2DM). Studies indicate that these two conditions mutually influence one another, complicating disease management and elevating the risk of several chronic diseases, including cardiovascular disease, diabetes-related microvascular complications, chronic kidney disease, and autonomic neuropathy (8, 9). It is well-known that T2DM is primarily characterized by inadequate insulin secretion and insulin resistance (IR), which arise from a combination of genetic and environmental factors (10). IR plays a central role in the pathogenesis of T2DM and is closely associated with obesity. Studies have shown that patients with both NAFLD and T2DM often suffer from obesity, hyperlipidemia, and increased body mass index (11). In obese individuals, excessive free fatty acids are converted into triglycerides in the liver, leading to hypoxia in adipocytes and chronic inflammation that affects insulin target cells, thereby inhibiting insulin signaling and contributing to IR (11). IR can lead to hyperinsulinemia, which diminishes lipid utilization and promotes lipid synthesis in the liver. Consequently, patients with NAFLD frequently exhibit IR, heightening their risk of developing diabetes (11). Therefore, IR may represent a common pathophysiological mechanism linking T2DM and NAFLD (12). On the other hand, studies employing magnetic resonance imaging to assess hepatic steatosis and cirrhosis have revealed a high prevalence of NAFLD and advanced fibrosis among diabetic patients (13). This evidence shows the interconnectedness of diabetes and NAFLD, which collectively impact patient prognosis. A clinical study investigated the risk of cirrhosis and hepatocellular carcinoma in patients with NAFLD, identifying diabetes as an independent predictor of adverse liver outcomes (14). Furthermore, the presence of NAFLD in diabetic patients is associated with an increased risk of secondary complications such as retinopathy, nephropathy, and cardiovascular disease (CVD), thereby contributing to higher mortality rates (8). Therefore, the interaction between T2DM and NAFLD accelerates disease progression and heightens the risk of adverse events in both hepatic and extrahepatic tissues and organs. Although emerging studies suggest that NAFLD may elevate the risk of T2DM (15), there remains a notable lack of therapeutic strategies for NAFLD and reliable evaluation indicators for assessing the risk of T2DM in affected patients. Above all, the development of a specific evaluation approach is essential for early identification, effective prevention, and treatment, ultimately improving patient outcomes.

Uric acid (UA) is the final product of purine metabolism, specifically from adenine and guanine in humans. Hyperuricemia can occur when UA production is excessive or renal excretion of UA is diminished. This condition not only contributes to the development of cardiovascular diseases and chronic kidney disease but may also accelerate the progression of these disorders (16). Studies have demonstrated that UA can promote inflammatory responses and oxidative stress (17). High-density lipoprotein cholesterol (HDL-C), primarily synthesized in the liver, is recognized as an anti-atherosclerotic lipoprotein that facilitates the transport of cholesterol from extrahepatic tissues to the liver for metabolism, serving as a protective factor against CVD (18). Emerging studies have indicated that the uric acid to high-density lipoprotein cholesterol ratio (UHR) may function as an emerging biomarker for assessing the body’s state of inflammatory and oxidative stress (19). Furthermore, UHR has been closely associated with various vascular and metabolic diseases, including coronary heart disease, NAFLD, and T2DM (20, 21). In addition to serum biomarkers, our previous study and others demonstrated that electrocardiography (ECG) is a non-invasive and convenient method for cardiac assessment, with certain ECG parameters serving as biomarkers to evaluate the severity and prognosis of coronary heart disease and other cardiovascular and cerebrovascular disorders (22–26). In light of these findings, the primary objective of this study is to explore the association between UHR and ECG parameters in patients with NAFLD and T2DM, as well as to investigate the clinical predictive value of these biomarkers in predicting the risk of T2DM in individuals with NAFLD, thereby providing a scientific basis for the disease prevention and treatment.

## 2 Materials and methods

### 2.1 Study subjects

Two hundred and fifteen newly diagnosed NAFLD patients admitted to the short-term rehabilitation center of our hospital from July 2023 to October 2024 were included as subjects for this study. Subjects were categorized into two groups based on the presence of T2DM: the NAFLD with T2DM group (NAFLD-T2DM group, n=102) and the NAFLD without T2DM group (NAFLD-nT2DM group, n=113). The diagnosis of NAFLD was based on the typical imaging changes underwent a liver ultrasound which included (1): increased near-field ultrasound beam in the liver (2), attenuated far-field ultrasound beam in the liver (3), unclear display of intrahepatic structures, after excluding other forms of liver disease (27). For the diagnosis of T2DM, the patients met the criteria established by the American Diabetes Association (28). Exclusion Criteria (1): Type 1 diabetes or secondary diabetes (2). Malignant tumors, severe infectious diseases, or acute complications of diabetes (3). Male subjects with an ethanol intake of ≥140 g per week, or those with other severe liver diseases (such as drug-induced liver injury, viral hepatitis, liver cirrhosis, liver cancer, autoimmune hepatitis, etc.) (4). Patients with severe damage to the heart, brain, kidneys, or other organs (5). Patients currently using medications that affect blood lipids, uric acid, or hepatic steatosis. This study received approval from the hospital ethics committee (WTSLL2024007) and was registered with the National Medical Research Registry System (MR-32-24-051272). All participants provided informed consent.

### 2.2 Methods

#### 2.2.1 Data collection and measurement

After 10 minutes of seated rest, all participants had their systolic and diastolic blood pressures measured using a standard mercury sphygmomanometer. Both groups of participants fasted for 8–10 hours, and 5 mL of venous blood was collected from each participant the following morning. An automatic biochemical analyzer was used to measure the levels of triacylglycerol (TG), total cholesterol (TC), aspartate aminotransferase (AST), alanine aminotransferase (ALT), serum creatinine (Scr), uric acid (UA), fasting plasma glucose (FPG), low-density lipoprotein cholesterol (LDL-C), high-density lipoprotein cholesterol (HDL-C), apolipoprotein A1 (ApoA1), apolipoprotein B (ApoB), lipoprotein (a) (Lpa), cystatin C (Cys-C), and small dense LDL cholesterol (sdLDL-C). The UHR was determined as the ratio of UA to HDL-C.

#### 2.2.2 ECG examination

An ECG examination was performed using the MAC800 ECG machine from GE Healthcare. Participants were instructed to lie in a supine position with their chests fully exposed. The skin was cleaned with an alcohol swab. After the participants were asked to breathe calmly, the ECG was recorded at a paper speed of 25 mm/s and a gain of 10 mm/mV. The analysis of the ECG results was conducted by a professional electrocardiographer with intermediate or higher qualifications.

#### 2.2.3 Statistical analysis

Data were analyzed using SPSS 25.0 software. For normally distributed continuous data, results were expressed as mean ± SD, and comparisons between the two groups were conducted using the independent samples t-test. For continuous data that did not follow a normal distribution, results were presented as a median and interquartile range, and comparisons between groups were performed using the Mann-Whitney U test. Categorical data were expressed as frequencies, and comparisons were made using the chi-square test. Variables that demonstrated statistical significance in the univariate analysis were further analyzed using multivariate logistic regression analysis. Receiver operating characteristic (ROC) curves were generated using MedCalc 22.0 software to assess the predictive value of each indicator for NAFLD combined with T2DM. A value of P < 0.05 was considered statistically significant.

## 3 Results

As shown in **Table 1**, we initially compared the general and biochemical data between the two groups of patients. Among the 215 participants, 102 were classified in the NAFLD-T2DM group, with a mean age of 63.87 ± 4.43 years, and 113 were in the NAFLD-nT2DM group, with a mean age of 63.59 ± 4.90 years. No statistically significant differences were observed between the two groups regarding age, systolic blood pressure (SBP), diastolic blood pressure (DBP), TC, LDL-C, HDL-C, ApoA1, ApoB, Lpa, Cys-C, sdLDL-C, ALT, and Scr. However, levels of TG, FPG, UA, and UHR were significantly higher in the NAFLD-T2DM group compared to the NAFLD-nT2DM group (P < 0.05). Conversely, the level of AST was significantly lower in the NAFLD-T2DM group (P < 0.05).

**Table 1:** Comparison of the baseline characteristics in two groups.

Feature	NAFLD-T2DM (n=102)	NAFLD-nT2DM (n=113)	t/z value	P value
Age (year)	63.87 ± 4.43	63.59 ± 4.90	-0.094	0.925
SBP (mmHg)	138.08 ± 18.59	133.63 ± 16.04	-0.538	0.591
DBP (mmHg)	85.82 ± 8.59	86.30 ± 8.78	0.306	0.760
TG (mmol/L)	2.00 (1.23,3.40)	1.47 (1.13,2.18)	-2.183	0.029
TC (mmol/L)	4.65 ± 1.00	4.72 ± 1.05	0.395	0.693
LDL-C (mmol/L)	3.06 ± 0.72	3.08 ± 0.79	0.366	0.715
HDL-C (mmol/L)	1.27 ± 0.23	1.36 ± 0.29	1.218	0.225
Apo A1 (g/L)	1.33 ± 0.21	1.42 ± 0.23	1.221	0.224
Apo B (g/L)	1.00 (0.77,1.13)	0.88 (0.75,1.05)	-1.763	0.078
Lpa (mg/L)	78 (38.75,245.25)	85 (38.25,170.75)	-0.446	0.655
Cys-C (mg/L)	1.10 (0.96,1.28)	1.12 (0.99,1.26)	-0.270	0.787
sdLDL-C (mg/L)	452.32 ± 129.77	429.85 ± 114.35	-0.494	0.622
FPG	9.14 ± 1.69	5.79 ± 0.60	-18.863	0.000
AST (U/L)	22 (17.68,28.88)	25.55 (21.83,32.43)	-2.758	0.006
ALT (U/L)	23.3 (15.05,30.35)	24.4 (19.10,31.50)	-1.570	0.116
Scr (μmol/L)	67.95 (59.98,8.50)	74.15 (65.15,82.93)	-1.719	0.086
UA (μmol/L)	392.00 (345.00,455.25)	380.00 (326.65,451)	-2.231	0.026
UHR	300.16 (251.16,364.75)	287.13 (226.74,355.84)	-3.069	0.002

Data were presented as mean ± SD or medium. NAFLD-T2DM, non-alcoholic fatty liver disease (NAFLD) with type 2 diabetes mellitus (T2DM); NAFLD-nT2DM, NAFLD without T2DM; SBP, systolic blood pressure; DBP, diastolic blood pressure; TG, triacylglycerol; TC, total cholesterol; LDL⁃C, low-density lipoprotein cholesterol; HDL⁃C, high-density lipoprotein cholesterol; ApoA1, Apolipoprotein A1; ApoB, apolipoprotein B; Lpa, lipoproteina; Cys-C, cysatinC; sdLDL-C, small dense low-density lipoprotein-cholesterol; FPG, fasting plasma glucose; AST, aspartate aminotransferase; ALT, alanine aminotransferase; Scr, serum creatinine; UA, uric acid; UHR, uric acid to high-density lipoprotein cholesterol ratio.

Subsequently, we analyzed the incidence of ECG abnormalities in both groups. The incidence of abnormal ECG (specifically ST-T changes) was significantly higher in the NAFLD-T2DM group compared to the NAFLD-nT2DM group (P < 0.05) (**Table 2**). However, no statistically significant differences were found between the two groups regarding the incidence of sinus tachycardia/bradycardia, first degree atrioventricular block, left ventricular high voltage, incomplete/complete right bundle branch block, premature beats, abnormal Q wave, QT interval prolongation, and left/right atrial abnormalities (P > 0.05) (**Table 2**). Furthermore, we compared ECG parameters between the two groups. As shown in **Table 3**, the heart rate and P wave duration were significantly higher in the NAFLD-T2DM group, while the QT interval was shorter (P < 0.05). These findings indicate that characteristic ECG changes are present in patients with NAFLD combined with T2DM.

**Table 2:** Comparison of the incidence of abnormal electrocardiograms in two groups.

Parameters	NAFLD-T2DM (n=102)	NAFLD-nT2DM (n=113)	χ^2^ value	P value
Sinus tachycardia/bradycardia	17 (16.7)	16 (14.2)	0.19	0.663
First degree atrioventricular block	9 (8.8)	5 (4.4)	1.493	0.222
Left ventricular high voltage	11 (10.8)	12 (10.6)	0.001	0.972
Incomplete/complete right bundle branch block	10 (9.8)	9 (8.0)	0.188	0.664
Premature beat	3 (2.9)	2 (1.8)	0.309	0.578
Abnormal Q wave	11 (10.8)	9 (8.0)	0.419	0.518
ST-T changes	47 (46.1)	30 (26.5)	4.195	0.041
QT interval prolongation	7 (6.9)	5 (4.4)	0.54	0.462
Left/right atrial abnormalities	4 (3.9)	2 (1.8)	1.071	0.301

Data were presented as count (%). NAFLD-T2DM, non-alcoholic fatty liver disease (NAFLD) with type 2 diabetes mellitus (T2DM); NAFLD-nT2DM, NAFLD without T2DM.

**Table 3:** Comparison of electrocardiogram parameters in two groups.

Parameters	NAFLD-T2DM (n=102)	NAFLD-nT2DM (n=113)	t/z value	P value
Heart rate (beats/minute)	75 (67.75,83)	69 (62,76.75)	-3.559	0.000
PR (ms)	164.37 ± 21.11	163.3 ± 20.21	-0.328	0.743
QRS (ms)	91 (83.5,98)	90 (84,96)	-0.035	0.972
QT interval (ms)	383.32 ± 26.27	393.38 ± 28.96	2.267	0.025
QTc (ms)	422.77 ± 21.32	417.71 ± 21.75	-1.480	0.141
RV5 (mv)	1.66 ± 0.52	1.71 ± 0.58	0.576	0.565
RV5+SV1 (mv)	2.35 ± 0.75	2.40 ± 0.79	0.393	0.695
P wave duration (ms)	118 (113,120)	106 (103,108)	-8.937	0.000

Data were presented as mean ± SD or medium. NAFLD-T2DM, non-alcoholic fatty liver disease (NAFLD) with type 2 diabetes mellitus (T2DM); NAFLD-nT2DM, NAFLD without T2DM.

To elucidate the factors associated with the coexistence of NAFLD patients with T2DM, we conducted a multivariate logistic regression analysis. As shown in **Table 4**, the analysis included parameters that demonstrated statistical significance in the univariate analysis: TG, AST, UA, UHR, ST-T changes, heart rate, QT interval, and P wave duration as independent variables, with the presence of T2DM in NAFLD as the dependent variable. The results indicated that UHR, ST-T changes, heart rate, QT interval, and P wave duration are independent influencing factors for the presence of T2DM in NAFLD (**Table 4**). This suggests that these indicators are associated with the coexistence of T2DM in patients with NAFLD and may serve as risk indicators for the development of T2DM in this population.

**Table 4:** Logistic regression analysis of the indicators in NAFLD with T2DM.

Parameters	B	SE	Wald value	OR	95% CI	P value
TG	0.063	0.149	0.179	1.065	0.795~ 1.428	0.672
AST	-0.045	0.029	2.455	0.117	0.904~ 1.011	0.956
UA	0.003	0.005	2.455	1.003	0.993~ 1.012	0.051
UHR	0.001	0.005	0.023	1.001	0.991~ 1.011	0.031
ST-T changes	2.381	0.628	14.385	10.814	3.160~ 37.010	0.000
Heart rate	0.067	0.045	2.174	1.069	0.978~ 1.168	0.001
QT interval	-0.003	0.016	0.038	0.846	0.997~ 0.966	0.025
P wave duration	0.357	0.058	37.227	1.429	1.274~ 1.602	0.000

TG, triacylglycerol; AST, aspartate aminotransferase; UA, uric acid; UHR, uric acid to high-density lipoprotein cholesterol ratio; NAFLD, non-alcoholic fatty liver disease; T2DM, type 2 diabetes mellitus.

**Figure 1:**
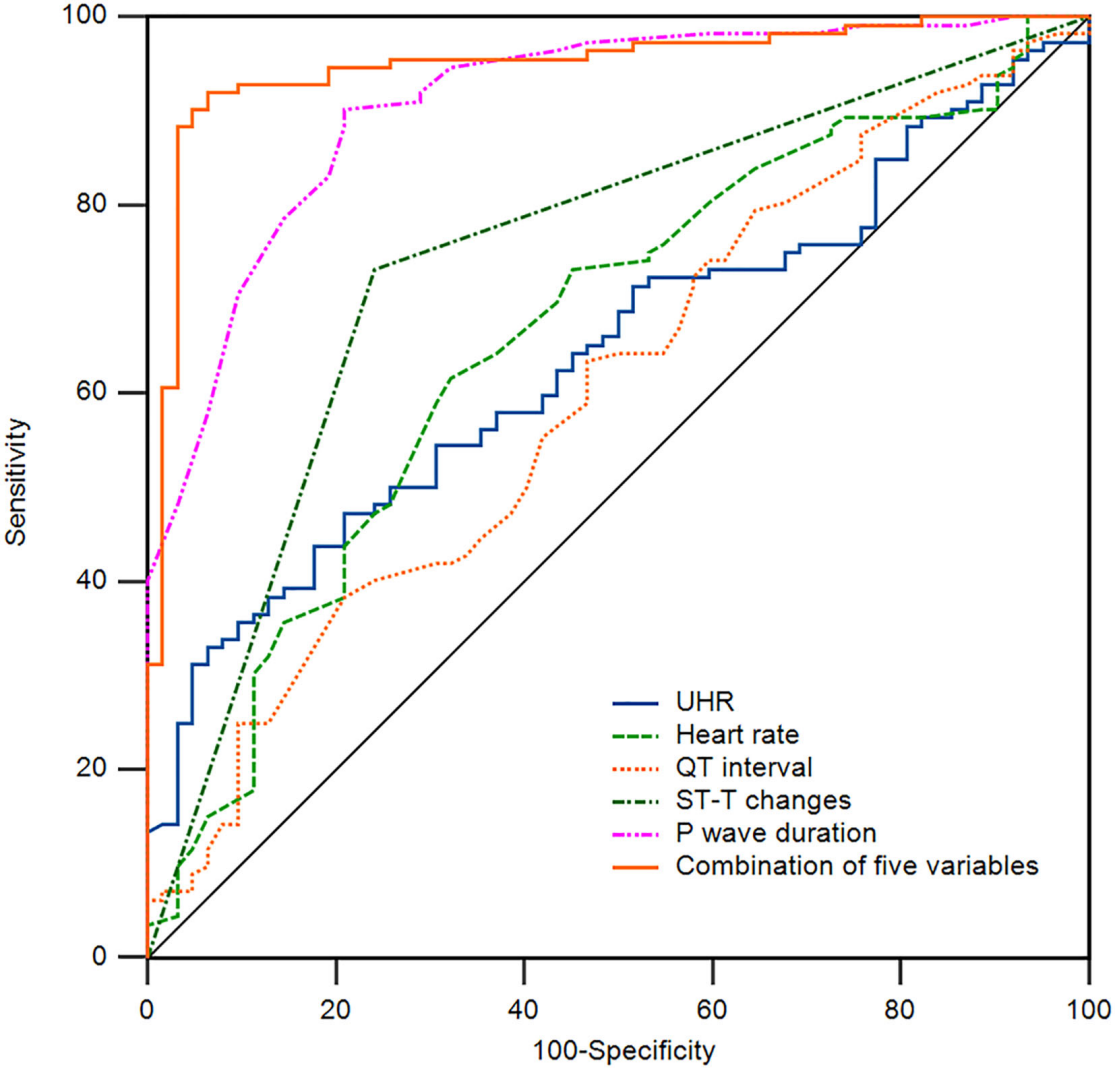
Receiver operating characteristic (ROC) curve of the indicators for the prediction of T2DM in NAFLD patients. The area under the curve (AUC) of UHR, Heart rate, QT interval, ST-T changes, P wave duration, and a combination of five variables for the prediction of T2DM in NAFLD patients are 0.641, 0.663, 0.602, 0.745, 0.909, and 0.949. NAFLD, non-alcoholic fatty liver disease; T2DM, type 2 diabetes mellitus; UHR, uric acid to high-density lipoprotein cholesterol ratio.

Additionally, we employed ROC curve analysis to assess the predictive value of the relevant indicators for NAFLD combined with T2DM. We conducted ROC curve analyses for UHR, heart rate, QT interval, ST-T changes, P wave duration, and the combination of five variables, calculating the area under the curve (AUC). As shown in **Table 5** and **Figure 1**, the AUC for predicting NAFLD combined with T2DM using UHR, heart rate, QT interval, ST-T changes, P wave duration, and the combination of five variables were 0.641 (95% CI: 0.565-0.712, P < 0.05), 0.663 (95% CI: 0.588-0.733, P < 0.05), 0.602 (95% CI: 0.525-0.675, P < 0.05), 0.745 (95% CI: 0.674-0.808, P < 0.05), 0.909 (95% CI: 0.856-0.947, P < 0.05), and 0.949 (95% CI: 0.905-0.977, P < 0.05), respectively. The AUC for the combination of five variables diagnosis was significantly higher than that for UHR, heart rate, QT interval, ST-T changes, and P wave duration, with statistical significance (*Z* = 7.087, *Z* = 6.227, *Z* = 7.358, *Z* = 5.297, *Z* = 1.513, P < 0.05). Moreover, the sensitivity and specificity of the combination of five variables for predicting T2DM in NAFLD were 91.96% and 93.55% (**Table 5**), respectively, which were significantly higher than those of single indicators, indicating a superior predictive value.

**Table 5:** Analysis of the indicators in predicting NAFLD with T2DM.

Parameters	AUC	Truncation value	Sensitivity	Specificity	95% CI	*P* value
UHR	0.641	246.77	33.02	93.5	0.565~0.712	0.000
Heart rate	0.663	71bpm	61.61	67.74	0.588~0.733	0.000
QT interval	0.602	402ms	38.39	79.03	0.525~0.675	0.021
ST-T changes	0.745	–	73.21	75.81	0.674~0.808	0.000
P wave duration	0.909	112ms	90.18	79.03	0.856~0.947	0.000
Combination of five variables	0.949	–	91.96	93.55	0.905~0.977	0.000

UHR, uric acid to high-density lipoprotein cholesterol ratio; NAFLD, non-alcoholic fatty liver disease; T2DM, type 2 diabetes mellitus.

## 4 Discussion

NAFLD is a significant contributor to chronic liver diseases. In light of the current absence of effective therapeutic strategies, the prevention and management of this condition have emerged as critical scientific challenges. This study examined patients undergoing short-term convalescence in our hospital. We found that, compared to NAFLD patients without T2DM, serum markers such as the UHR and ECG-related parameters exhibited distinctive changes in NAFLD patients. These changes may serve as indicators of the risk of developing T2DM in this population. Furthermore, the combination of UHR and ECG-related parameters—including ST-T changes, heart rate, QT interval, and P wave duration demonstrated enhanced sensitivity and specificity in predicting the risk of T2DM in NAFLD patients, suggesting their potential as biomarkers.

It is important to note that simple NAFLD, despite its relatively minor hepatic injury, increases the risk of various metabolic disturbances, including IR, T2DM, dyslipidemia, and hypertension (1, 29). Studies have shown that elevated levels of transaminases are positively correlated with the future risk of developing T2DM, with NAFLD patients facing more than double the risk (30, 31). Additionally, it is estimated that 37% of T2DM patients with NAFLD will develop non-alcoholic steatohepatitis (NASH) globally (32).

Currently, the precise pathogenesis of NAFLD in conjunction with T2DM remains incompletely understood. However, existing studies suggest that its development is closely linked to inflammatory responses. This relationship may arise from the regulatory effects of inflammatory cytokines on insulin sensitivity, which can trigger IR and ultimately disrupt the metabolic equilibrium of the liver (11). IR, a common pathological basis for both metabolic disorders, impairs the liver’s ability to suppress hepatic glucose production in response to insulin. Concurrently, it promotes *de novo* lipogenesis through the activation of the Notch signaling pathway. The accumulation of fat in the liver results from impaired processes of free fatty acid (FFA) uptake, synthesis, export, and oxidation. In patients with NAFLD, the degree of hepatic steatosis correlates with elevated plasma levels of free FFA, attributable to the impaired capacity of adipose tissue to inhibit lipolysis (33). FFA can activate the c-Jun N-terminal kinase (JNK) signaling pathway, inducing cellular stress responses, inflammation, apoptosis, and mitochondrial dysfunction. Furthermore, the JNK signaling pathway can lead to the phosphorylation of peroxisome proliferator-activated receptor gamma, exacerbating lipotoxicity and hepatic inflammatory responses (34). IR results in decreased sensitivity of target organs to insulin, further deteriorating glucose metabolism and advancing disease progression (35). Studies indicate that UA plays a pro-inflammatory role in various chronic diseases. Elevated UA levels can induce oxidative stress, resulting in IR in pancreatic β-cells (36). IR can also lead to decreased HDL-C concentrations through multiple mechanisms. On one hand, under IR conditions, reduced lipoprotein lipase activity decreases the hydrolysis of triglycerides in chylomicrons and very low-density lipoproteins, further limiting HDL-C production. On the other hand, increased hepatic lipase activity enhances the clearance of HDL-C (37). Consequently, the UHR can more effectively predict the extent of metabolic disorders. Clinical studies have confirmed that UHR is closely associated with various metabolic diseases, including diabetes, diabetic nephropathy, cardiovascular diseases, and metabolic-associated fatty liver disease (38, 39). Studies showed that UHR serves as a useful predictor of glycemic control in men with T2DM, as it is positively correlated with glycated hemoglobin (HbA1c) and fasting plasma glucose levels (39). Zhang suggested that UHR is independently associated with an increased risk of NAFLD, with their study indicating that a 1% increase in UHR level corresponds to a 10.5% increased risk of developing NAFLD (38). UHR was elevated in metabolic syndrome and is suggested to possess greater sensitivity and specificity than other criteria used to identify subjects with metabolic syndrome (40).

CVD is the leading cause of mortality among patients with T2DM, accounting for two-thirds of all-cause deaths (41). Elevated levels of HbA1c are closely associated with an increased risk of heart disease and overall mortality (42). Various glycemic control strategies, particularly those focusing on weight reduction, effectively reduce the likelihood of major adverse cardiovascular events, thereby confirming the efficacy of diabetes treatment in yielding positive cardiovascular outcomes (43). In addition to macrovascular complications associated with CVD, patients with NAFLD also face an elevated risk of microvascular disease, particularly chronic kidney disease, which is strongly correlated with CVD and T2DM (44). Hypertension, T2DM, smoking, and physical inactivity were significantly associated with primary and secondary ECG abnormalities (44). The incidence of primary abnormalities increased significantly with the number of cardiovascular risk factors. One study emphasized the importance of implementing preventive measures for primary and secondary ECG abnormalities, which may indicate an increased risk of cardiovascular disease (45). Another study investigating atrial electromechanical delay (EMD) and P wave dispersion (Pd) in patients with T2DM found that, compared to healthy volunteers, patients with T2DM exhibited significantly higher EMD and Pd, with interatrial EMD positively correlated with Pd and left atrial volume index (46). This suggests that T2DM may increase the risk of atrial fibrillation (AF) by affecting atrial conduction properties, underscoring the necessity of regular cardiac monitoring in patients with T2DM (46). The study explored the mechanisms underlying prolonged P wave duration in rats with T2DM, revealing that the prolonged P wave duration in T2DM rats was not related to left atrial size but was instead caused by abnormalities in atrial myocyte ion currents and the expression of gap junction proteins Cx40 and Cx43, as well as fibrosis of the atrial tissue. The results indicated that T2DM may lead to prolonged P-wave duration by affecting the expression of Cx40 and Cx43 proteins and inducing atrial fibrosis (47). Other studies have found that, compared to healthy controls, patients with T2DM initially experience an increase in heart rate during hypoglycemia; however, after one hour of sustained hypoglycemia, the heart rate decreases to baseline levels, accompanied by reactivation of vagal tone (48). Moreover, during hypoglycemia, T2DM patients exhibit shorter QT intervals, QTc intervals, and T-wave amplitude and symmetry compared to the control group, demonstrating more pronounced cardiac repolarization abnormalities (48). This is consistent with the findings of this study, which discovered that the group with NAFLD combined with T2DM had an increased heart rate, shortened QT interval, and prolonged P wave duration, indicating a heightened likelihood of cardiac repolarization abnormalities. However, continuously monitored blood glucose and ECG in patients with type 1 diabetes showed that during hyperglycemia, the QTc interval was, on average, longer and positively correlated with the duration of hyperglycemia (49). Additionally, neuropathy was significantly associated with a prolonged QTc interval, emphasizing the potential importance of continuous monitoring of blood glucose and ECG data in clinical practice (49). On the other hand, compared to healthy controls, T2DM patients had significantly prolonged T-peak to T-end (Tp-e) intervals, Tp-e/QT ratios, and Tp-e/QTc ratios, indicating an increased risk of ventricular arrhythmias (50). Moreover, there was no significant statistical association between T-wave alteration (TWA) and T2DM after adjusting for confounding factors such as age, gender, and hypertension, although the incidence of T-wave abnormalities was higher in diabetic patients (51). However, TWA is independently associated with changes in left ventricular myocardial structure, impaired left ventricular systolic function, and left ventricular diastolic dysfunction, suggesting that TWA may serve as a useful indicator for identifying early myocardial structural and functional abnormalities in T2DM patients (52). Similarly, the prevalence of ST-T changes in patients with NAFLD was 6.5%, and among participants with ST-T changes, the prevalence of NAFLD was 42.9%, suggesting a significant independent association between ST-T changes and NAFLD (53). This study also confirmed an increased incidence of ST-T changes in the group with NAFLD combined with T2DM, indicating the need for the early assessment of cardiovascular disease risk (53). Patients with hemodynamically significant coronary lesions had significantly increased UA levels (54). Higher UA levels had a significantly higher probability of cardiac conduction block (mainly first-degree atrioventricular block) than those with normal UHR levels (55). This association remained significant even after adjusting for multiple cardiovascular risk factors and potential confounding variables, suggesting that elevated UA may be an independent predictor of cardiac conduction block in T2DM patients (55). This indicates a strong association between UHR levels and ECG parameters. The logistic regression analysis in this study revealed that UHR, ST-T changes, heart rate, QT interval, and P wave duration are all independent influencing factors for the development of T2DM in patients with NAFLD. The results of ROC curve analysis showed that the AUC for the combination of five variables was 0.949 (95% CI: 0.905–0.977, P < 0.05), with a sensitivity of 91.96% and a specificity of 93.55%, which is significantly higher than that of any single indicator. This combination provides better predictive value for patients with NAFLD who develop T2DM and offers a valuable reference for clinical practice. Therefore, in clinical settings, especially in NAFLD patients with glucose/lipid dysfunction as well as obesity, there should be an emphasis on regularly monitoring and assessing UHR, ST-T changes, heart rate, QT interval, and P wave duration. Furthermore, UHR and ECG testing are commonly utilized and cost-effective in the real-word medical settings. Collectively, this approach contributes to the protection of the cardiac health in patients, thereby improving their quality of life.

However, the current study has several limitations, including cross-sectional and single-center (rehabilitation-center setting), which preclude the establishment of a causal relationship between UHR and ECG parameters and the development of T2DM in NAFLD patients. Furthermore, this study did not include an analysis of potential factors such as body mass index (BMI), medication, and lifestyle of the subjects. To generalize the findings to broader and more diverse populations, future studies are needed to validate these findings by increasing the sample size, enhancing multicenter collaboration, and improving follow-up protocols.

In summary, UHR levels and ECG parameters are significant factors associated with the risk of T2DM in NAFLD. The combination of UHR and ECG parameters exhibits predictive value for the presence of T2DM in this population. UHR and ECG changes in patients with NAFLD and T2DM should be closely monitored in the clinical setting. Furthermore, it is essential to integrate the strengths of multiple disciplines, such as hepatology and endocrinology, to provide comprehensive management for patients with these comorbidities. This approach will facilitate the establishment of a more precise and detailed tiered diagnosis and treatment system, thereby promoting effective management of patients with these comorbid conditions.

## Data Availability

The original contributions presented in the study are included in the article/supplementary material. Further inquiries can be directed to the corresponding author.
